# Is the Bacteriological Theory of Disease Waning?

**Published:** 1896-04

**Authors:** 


### Is the Bacteriological Theory of Disease Waning?

It is an old saying "that every dog has his day," and it seems to be equally true of medical theories. We have observed, during the last year, that much has been said by the most eminent of authorities tending to express doubt as to the invulnerability of the germ doctrine as the sole cause of disease. The ultra germ theorists are dropping out of sight and allowing the more rational facts of climatic and atmospheric changes as producers of disease full sway in the theories of the present day.

It was not long since a simple catarrhal inflammation of the nasal passages, commonly called a "cold," was considered the result of bacillary invasion. We now are called upon to consider the influences exerted by draughts, sudden changes in atmospheric temperature and other unhygienic conditions, reverting to the former days when these surrounding elements were looked upon as the sole authors of disease.

But our forefathers were not always so far out of the way in their reasoning, and there were no greater fools in a century back than sometimes exhibit themselves at the present time. The one great failing with the individual members of the profession of the nineteenth century is the inordinate desire for notoriety and the consequent tendency it exerts for rushing forward with every new theory until it becomes a veritable fad.

Fads are often instructive and usually safe enough if not carried to excess. So it is with the germ doctrine. We have bacilli and bacilli—innumerable quantities of them—growing in such situations as best support them by favorable environments.

Probably many of them produce toxines. Undoubtedly many are harmless. Possibly some may even be termed curative by their antagonism for those that are virulent. But all this does refute the fact that the bacillus, of whatever genus he may be, cannot find in the purely healthy human blood-cell a favorable field for his cultivation and growth. That must be sought for in the degenerated corpuscle.

We must study, then, the various causes which influence the degeneration of the blood-cell if we would get at the real prime mover in etiology. These are, undoubtedly, more or less controlled by the sensory nerves acting through outside stimuli and explains vastly better how the production of a "cold" may arise from draughts, bad air and warm winter storms.

It is, however, among the contagious diseases that the claims of the germ theory seem to have their strongest hold for recognition; but when one stops to think that pure air and thoroughly oxygenated blood are the best preventives for contagious diseases, it will be readily understood how we may find specific germs early in a given disease without their proving more than symptomatic phenomena.—*Times and Register.*

